# Preparation and Evaluation of Intravaginal Ring Containing Drospirenone

**DOI:** 10.1155/2013/192408

**Published:** 2013-01-17

**Authors:** Ying Zhang, Chun-Xiao Li, Mei-Ying Ning, Xue-Yan Duan, Ying Liu

**Affiliations:** ^1^Research Center of Biomaterial and Novel Drug Delivery Systems, National Research Institute for Family Planning, No. 12 Da Hui Si Haidian District, Beijing 100081, China; ^2^Chinese Academy of Medical Science and Peking Union Medical College, No. 9, Dongdan No. 3 Avenue, Beijing 100730, China

## Abstract

In the present study, we investigated the feasibility of the vaginal administration of drospirenone silicone IVR. The in vitro release characteristics of matrix-type and reservoir-type IVR were compared under sink conditions in 21 days. At the same time, API excipients compatibility and preformulation study was performed by HPLC, IR, and DSC methods. Biocompatibility of reservoir system was evaluated by tolerability on tissue level in rats. It was found that, under strong light exposure, high temperature, and high humidity conditions, drospirenone and excipients had no significant interactions. The daily release of reservoir-type IVR was about 0.5 mg/d sustaining 21 days, which significantly decreased the burst effect compared with the matrix system. When drospirenone was modified by the PVPk30 in the reservoir system formulation, the daily release rate increased to 1.0 mg/d sustaining 21 days. The cumulative release of reservoir-type IVR was fitted to zero release equation. In addition, biocompatibility of drospirenone IVR system in this dosage is safe. It is feasibility feasibile to further developed for safe, convenient, and effective contraceptive drug delivery with reduced dosing interval.

## 1. Introduction

**Figure 1:**
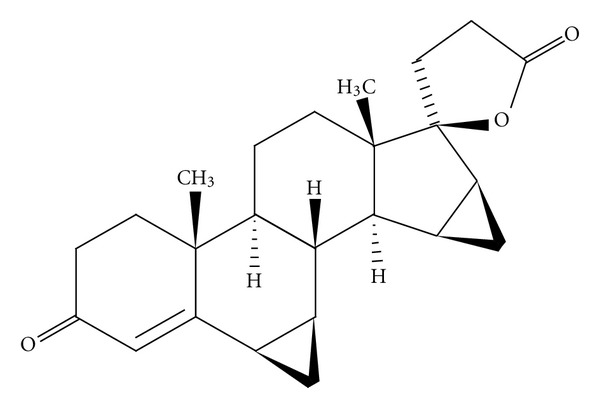
Drospirenone.

Drospirenone (6b,7b,15b,16b-dimethylene-3-oxo-17a-pregn-4-ene-21,17-carbolactone) is an analogue of the antimineralocorticoid spironolactone (Figure 1) that is synthesized from androstenolone. Unlike other progestogens, drospirenone, an analogue of spironolactone, has biochemical and pharmacologic profiles similar to endogenous progesterone, especially regarding antimineralocorticoid and antiandrogenic activities. Drospirenone counteracts the estrogen-induced stimulation of the renin-angiotensin-aldosterone system and blocks testosterone from binding to androgen receptors. Because of these characteristics, it has the potential to reduce body weight, blood pressure, and low-density lipoprotein levels and to enhance high-density lipoprotein levels. As a combination, oral contraceptive, drospirenone with ethinyl estradiol, is effective and has positive effects on weight and lipid levels [1].

Drospirenone has rapid oral absorption, its peak plasma concentration time is about 60–90 min, and the half-life is 30–35 h [2]. This product is to be administrated daily, otherwise it will affect the contraceptive effect. Therefore, sustained release of drug is preferred above the daily administration. Intravaginal rings (IVR) can serve as an alternative for the daily administration of tablets [3]. The contraceptive vaginal ring offers effective contraception that is self-administered, requires less frequent dosing than many other forms of contraception, and provides low doses of hormones. NuvaRing (Organon, Oss, The Netherlands), the contraceptive vaginal ring approved for use in the United States, contains etonogestrel and ethinyl estradiol. It is inserted into the vagina for 3 weeks, followed by 1-week ring-free period, and works by inhibiting ovulation. Most women note a beneficial effect on bleeding profiles and are satisfied with NuvaRing [3]. Serious adverse events are rare. In Chile and Peru, progesterone-only vaginal contraceptive rings are available for nursing women. Recently more studies are ongoing new formulations of intravaginal rings in the world [4–10].

In the present study we investigated the feasibility of drospirenone IVR formed by hot vulcanization method with silicone elastomer as the matrix polymer and encapsulated by the same kind of polymer.

## 2. Experimental

### 2.1. Materials

Drospirenone (Bat. 20101203) was provided by Zizhu Pharmaceutical Co. Ltd. Class VI elastomers (C6-165, Part A and Part B0005308947) was purchased from Dow Corning Corporation (USA). Polyvinylpyrrolidone_k30_ (PVP_k30_) were obtained from Fengli Jingqiu Commerce and Trade Co., Ltd. (Beijing, China). Hydrochloric acid, dehydrated alcohol, and sodium dihydrogen phosphate were obtained from Sinopharm Chemical Reagent Beijing Co., Ltd. (Beijing, China). Acetonitrile (HPLC grade) was purchased from Fisher Scientific (Fair Lawn, NJ, USA). All other chemicals and solvents were analytical reagents and obtained from local commercial source, SDS (Bat : L5750, Sigma).

### 2.2. Drospirenone Solubility in Different Release Medium

Overdose drospirenone which could keep saturated state in the following solvents was respectively placed into stoppered conical flasks containing 25 mL of a pure water, NaCL solution, PBS (pH 3.8), 0.03% SDS solution, and 0.3% SDS solution on magnetic stirrer (RT5PS25, IKA, Germany) at 37°C for 24 h. The samples were withdrawn, and subsequently the concentration of drug was assessed by UV-Vis spectrophotometric method.

### 2.3. Drospirenone Solubility in Silicone Elastomer [10]

Differential scanning calorimetry (DSC) was employed using a modulated instrument in which the total heat flow curve was not resolved into the reversing and nonreversing component. This curve was used to determine the solubility of drospirenone at its melting temperature in cured silicone elastomer. Drug-loaded silicone elastomer samples containing 1.0%, 2.0%, 5.0%, 10.0%, 15.0%, and 20.0% (w/w) drospirenone were moulded into sheets of 1.5 mm thickness with high temperature and high pressure, using Opening Mixing Mills (X(S)K-160, Shanghai wings of rubber and plastics machinery company limited, China). Sample cubes, approximately 5 mg in weight, were cut from the sheets and individually placed in weighed, matched (5 mg) hermetically sealed aluminium DSC test pans (Perkin-Elmer). Measurements were performed over the temperature range 40–250°C, at a heating rate of 20°C·min^−1^, so as to include the melting point of drospirenone (196–200°C). System calibration was verified using indium. The thermal properties were determined from the resulting DSC thermographs. The measurement of the area under the transition peak provides the direct base for the calculation of the energy associated with the phase transition. In this study the area was determined computationally and the areas determined were converted to the enthalpy of melting. At least three replicates of each sample were performed.

### 2.4. Preparation of Drospirenone-Loaded Sustained Silicone Intravaginal Rings

To improve the dissolution *in vitro* of drospirenone in silicon elastomer ring, solid dispersion of drospirenone was prepared by the conventional solvent evaporation method with polyvinylpyrrolidone (PVP_k30_) as carrier [11]. In this system, the solid dispersions of 0.3 : 1 and 1 : 3 wt/wt of drug to carrier were prepared. The mixture of drug and carrier was dissolved in ethanol. The solvent was evaporated under reduced pressure using a vacuum dryer at 70°C until complete evaporation. To ensure the residual solvent was completely removed, the solid mass was further dried in a vacuum oven at room temperature for 24 to 48 hours or until the weight constant was obtained. The resulting solid was pulverized and sieved. All of the samples were passed through a fine mesh (150 mm) and stored in desiccated environment until additional study.

Silicon elastomer C6-165 Part A and Part B was blended by Opening Mixing Mills to produce the silicon elastomer mix. The required amount of drospirenone or drospirenone solid dispersion was added to produce active mixture, and then the blends were removed from mixing mills and moulded in the circle mould (made in-house) at 130°C by flat vulcanizing machine (QLB-25D/Q, Shanghai wings of rubber and Plastics Machinery Company Limited, China) for 10 min to produce matrix Drospirenone IVRs. The matrix drospirenone IVR was coated with the same type silicon elastomer to form the reservoir system to control the release rate of the drug. 

### 2.5. *In Vitro* Drug Release Studies

*In vitro* release testing was performed in dissolution testing instrument (ZRS8G dissolution testing instrument, Tiandatianfa Scientific company, limited) according to the standard of dissolution methodology in Chinese Pharmacopeia (CP) (2010 Version, Apparatus II, rotating paddles, 50 rpm, 37°C, 200 mL of 0.3% SDS solution as medium). The cumulative release percentage was investigated in SDS buffer solution. Dissolution medium was renewed every 24 h (±30 min) over a 21-day period. Sampling followed by complete replacement of the release medium was performed every day for 21 days for each ring. Samples were stored at 4°C until detection.

The drug release was evaluated by UV-visible spectrophotometric method (PerkinElmer's Lambda 35 UV/vis systems, USA). Absorbance of the standard and sample solutions was measured at the wave length of 269 nm at the end of every time period. A linear calibration plot for drospirenone *A* = 0.0502C – 0.0084 was obtained over the range of 4.1–16.3 *μ*g·mL^−1^ (*r* = 1.000).

### 2.6. Stability of Drospirenone under Preparation Conditions

The thermal and the exposure light stability of drospirenone at the hot vulcanization method conditions was examined and analyzed for its nondegradability as follows: drospirenone was heated at 130°C and 140°C isothermally for 15 min under air atmosphere in an oven and was placed under LX 4500 strong light exposure 24 h (YG120 drug exposure testing instrument, Shanghai Experimental Instrument Factory Limited, China). Then, IR, DSC, and HPLC analyses were performed on the treated sample and compared to nontreated controls.

#### 2.6.1. Infrared Spectroscopy (IR)

IR spectra were recorded on a Nexus 670 FT-IR spectrophotometer (Thermo Nicolet, USA). Samples were prepared in KBr disks prepared with a hydrostatic press at a force of 5 T·cm^−2^ for 3 min. The scanning range was 4000–400 cm^−1^, the resolution was 4 cm^−1^, and scan times were 64.

#### 2.6.2. Differential Scanning Calorimetry (DSC)

Samples, approximately 2.5 mg in weight, were individually placed in weighed, sealed aluminium DSC test pans (Perkin-Elmer, USA). Each sample was run against a reference pan of air in standard mode under a nitrogen atmosphere. Measurements were performed over the temperature range 40–250°C, at a heating rate of 10°C·min^−1^ from 20°C, so as to include the melting point of drospirenone (196–200°C). The thermal properties were determined from the resulting DSC thermographs.

#### 2.6.3. High Performance Liquid Chromatography (HPLC)

Dissolve weighed quantity of drospirenone in acetonitrile, then dilute with mobile phase solvent to known concentration of about 2 mg/mL, and then mixe to be testing samples. Separately inject equal volumes of the samples, record the chromatograms, and keep track of the changes of impurities of nontreated and treated drospirenone. The HPLC condition and gradient elution program, respectively, are described in Tables 1 and 2.

### 2.7. Compatibility Study of Drospirenone and Excipients

Drospirenone and PVP_k30_, silicone rubber were performed the compatibility test, respectively, under the condition of 60°C, relative humidity of 92.5%, and illuminated at 4500 Lx respectively for 5 d and 10 d, at which time drug extraction and analysis were performed.

Changes of impurities from final treated silicon elastomer ring were extracted and detected by HPLC. The efficiency of the extraction method was about 85% as determined by the controls of blank ring spiked with a known amount of drospirenone. The test samples were dissolved in 2 mL of acetonitrile in a 50 mL centrifuge tube in triplicate followed by precipitation of the silicon elastomer by addition of 25 mL of cosolvent of water : acetonitrile = 1 : 1, v/v using a volumetric flask. The polymer was separated by centrifugation 2000 rpm for 5 min (Anke TDL-5, Ji'nan Qike equipment limited company, China), and the supernatant was analyzed for drospirenone imprurities change by HPLC. The caps of the centrifuge tubes were closed tightly and wrapped with Teflon tape during the process to minimize evaporation. The efficiency of the extraction of drospirenone from the silicon matrix using this method was determined by quantifying the extracted drospirenone content from controls of blank silicon elastomer pellets to which known amount of drospirenone was added. The mixtures of silicon elastomer pellets and drospirenone were then subjected to the same precipitation process and drospirenone as above. The concentration of final samples was controlled to be about 2 mg/mL.

### 2.8. Morphology Study of Vaginal Tissues after Application of Drospirenone IVR

In order to investigate the biological compatibility of IVR device, morphology study of vaginal tissues after application of drospirenone IVR was performed. Oophorectomized female Sprague-Dawley rats weighing 200 ± 10 g were used after a recovery period of at least 7 d. Drospirenone IVR cut into rod about 1 cm was implanted aseptically in preanesthetized animals. A midline laparotomy incision was made, and the vagina was isolated from surrounding soft tissues. The vaginal ring segment was inserted through the exterior vaginal opening. Insertion was performed as a clean procedure by trained technical staff. Once visualized by the surgeon, the implant was anchored with a 5-o Prolene suture to the vagina. The laparotomy incision was closed with absorbable suture and skin staples. The vaginal tissues of the sham surgery group (blank) treated rats (A) and drospirenone IVR rats (B) were isolated at seventh day and 21st day, fixed in 10% neutral carbonated-buffered formaldehyde, embedded in paraffin, and cut into slices. After hematoxylin-eosin staining, the slices were observed under a light microscope (×400).

## 3. Results and Discussion

### 3.1. Drospirenone Solubility in Release Medium

The solubilities in different solvent were investigated in order to select optimal release medium. The solubilities of drospirenone were determined to be 13.0 *μ*g/mL, 12.1 *μ*g/mL, 8.9 *μ*g/mL, 15.2 *μ*g/mL, and 392.6 *μ*g/mL in pure water, NaCL solution, PBS (pH 3.8), 0.03% SDS solution, and 0.3% SDS solution, respectively. According to sink conditions, the concentration of drospirenone in medium is 12 *μ*g/mL in 200 mL release medium if the probable daily maximum quantity of drug is 2.4 mg. Fivefold concentration is 60 *μ*g/mL which is far less than 392.6 *μ*g/mL. Therefore, 0.3% SDS solution was selected for release medium.

### 3.2. Drospirenone Solubility in Silicone Elastomer

DSC was employed to determine the solubility of drospirenone at its melting temperature in a silicone elastomer IVR device [12]. A DSC thermogram is depicted in Figure 3. No thermal changes were detected for silicone elastomer over the temperature range used. Characteristic endothermal transition peaks were observed for drug-elastomer formulations containing from 1% to 20% drospirenone.  Table 3 shows the thermal properties for the various drospirenone in silicone elastomer formulations at the 6 different loading levels. Thermograms obtained from the replicates of each formulation showed no noticeable alteration of the position and size of the transition peak, as evident from the low standard deviations for each formulation. In addition, low standard deviations were obtained for the peak and onset temperatures within each formulation, indicating good reproducibility.  Table 3 showed that Δ*H* (enthalpy of melting) increased linearly, with a corresponding increase in drospirenone loadings. An increase in the percentage concentration for incorporated drospirenone from 1% to 20% resulted in an increased trend in the peak temperature of the samples. The minimum peak temperature was observed for specimens containing 1% of drug (193.70 ± 0.20°C), whereas the maximum value was observed for specimens containing 20% drospirenone (195.07 ± 0.45°C). The enthalpy changes dΔ*H*), as measured by the area of the Drospirenone transition peak in the thermograms, were determined and used to quantify the amount of dissolved drug in silicone elastomer. A plot of Δ*H* versus drospirenone (%, w/w loading, *C*) was linear (Δ*H* = 48.588C − 0.0992) with a correlation coefficient of 0.9978. The straight line, however, did not pass through the origin. The intercept of the straight line with the *χ*-axis (Δ*H* = 0), which is the value at which no thermal response of drospirenone in the drug-elastomer system was recorded, coincides with the amount of drospirenone dissolved in the elastomer at the melting temperature of the drug. Thus, the silicone elastomer solubility of drospirenone according to the DSC method was found to be 2.042 mg/g, which could be helpful for interpreting IVR containing drug appearance and the drug release profile of IVR. Additionally, no exothermic peak was seen in any of the thermograms suggesting stability of drug loaded silicon elastomer matrix in the temperature range tested.

### 3.3. Appearance of IVR Made in-House

The final ring had the following characteristics: the matrix ring provided 5.0 mm ring cross-sectional diameter, 50.0 mm overall ring diameter, and 3.4 (±0.2) g mean weight of rings. The membrane thickness of reservoir ring was about 1.0 mm. The drospirenone incorporated polymer ring was not optically clearer (opalescent) than the blank silicon elastomer ring prepared by the same process without drug, suggesting that only part of drospirenone was dissolved in the polymer matrix. This phenomenon conforms to a result that the drug content of final optimized formulations was all far more than 2 mg/g that is drug solubility in silicone elastomer from the present solubility study.

### 3.4. Stability of Drospirenone under Preparation Conditions

In order to further study the possibility of drug degradation or oxidation, more information was gathered using FT-IR spectroscopy. Analysis on structure of drospirenone, it was assumed that there may be any possible changes. And this could lead to the peak broadening and shifting of the absorption bands of functional groups. The infrared spectra of nontreated drospirenone, 130°C and 140°C heated, and light exposure treated are presented in Figure 4. No changes comparing treated with nontreated samples were observed. The results indicate the stability of drospirenone under high temperature and strong light exposure.

**Figure 2:**
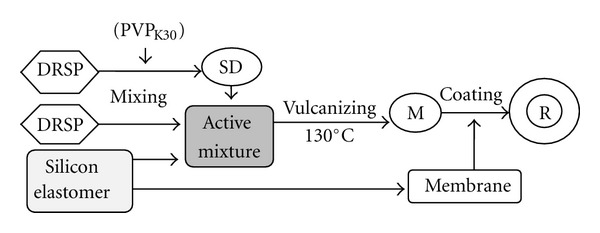
Flow chart of preparing IVR process. DRSP: drospirenone; SD: solid dispersion; M: matrix type; R: reservoir-type.

**Figure 3:**
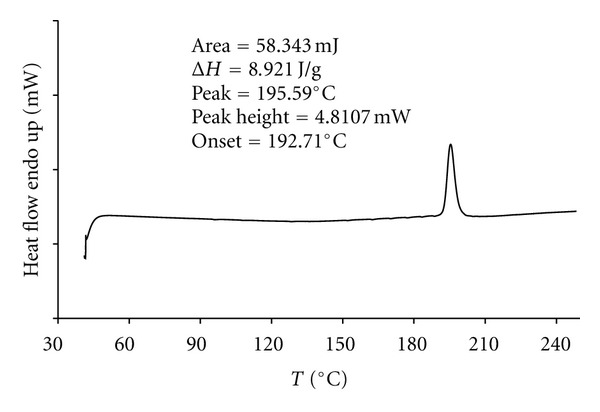
DSC spectrum of drospirenone under air atmosphere. An endothermic peak corresponding to melting point was obtained for drospirenone crystals at 195.59°C. No exothermic peak was seen in the thermograms suggesting stability of drospirenone-loaded silicone elastomer matrix in the temperature range tested.

**Figure 4:**
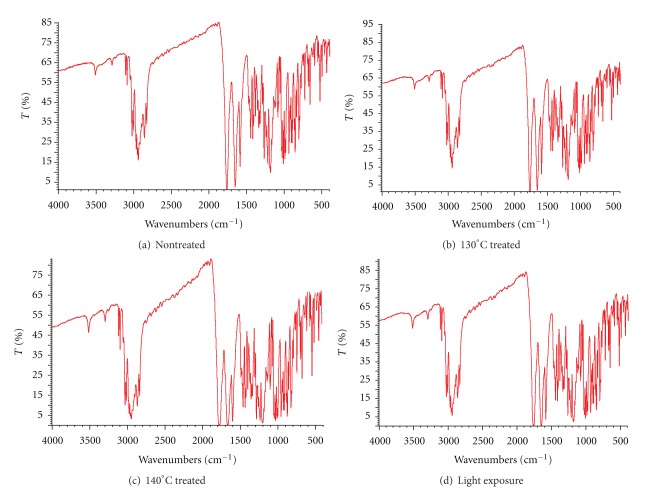
Infrared radiation scanning pattern of drospirenone compared nontreated with treated.

**Figure 5:**
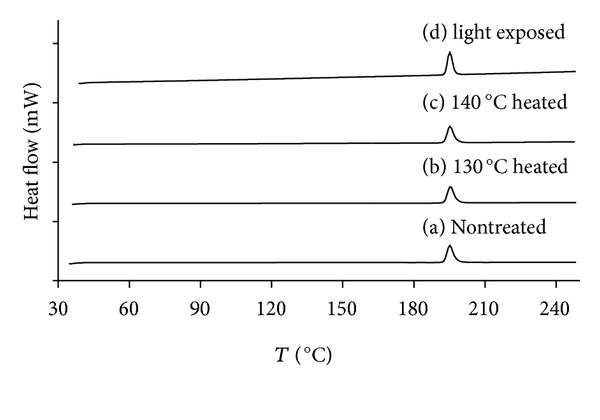
Differential scanning calorimetry thermogram of drospirenone in silicone elastomer compared nontreated with treated.

The DSC thermogram of drospirenone from 40 to 240°C showed no detectable degradation (Figure 5). The melting temperature of drospirenone was observed by an endotherm at 196°C. No exothermic peak was observed in the temperature ramp from 40 to 240°C (Figure 2) or in the isothermal step at 196°C (data not shown) indicating no detectable degradation of drospirenone under prepared conditions (130°C–140°C).

**Figure 6:**
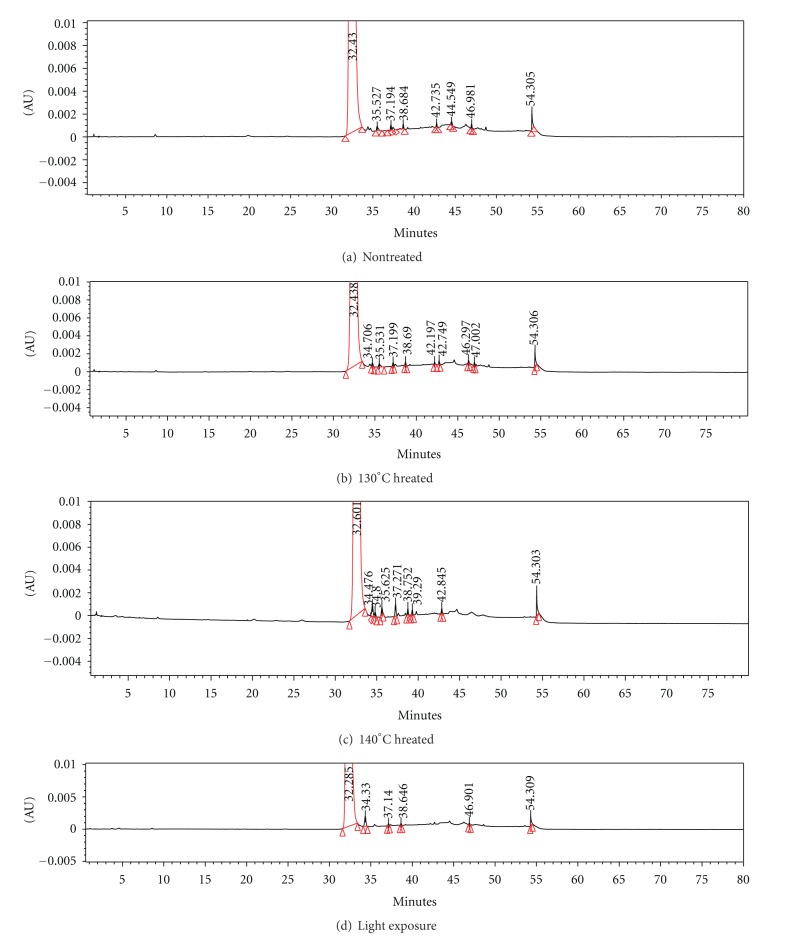
HPLC chromatogram of drospirenone compared nontreated with treated.

The stability of drospirenone was tested at preparing conditions (higher temperatures and strong light exposure conditions). No significant changes of quantities of impurities and no more new impurities in the HPLC spectra (see Figure 6) of the treated drospirenone sample were observed when compared with that of the nontreated samples.

All the above experiments results confirmed that drospirenone could be able to withstand high temperature and light exposure IVR process without degrading.

### 3.5. *In Vitro* Drug Release Studies

*In vitro* drug release testing was conducted in a dissolution apparatus, and release medium was 200 mL 0.3% SDS meeting with sink conditions, and release samples were determined by the UV-Vis spectrophotometry.

**Figure 7:**
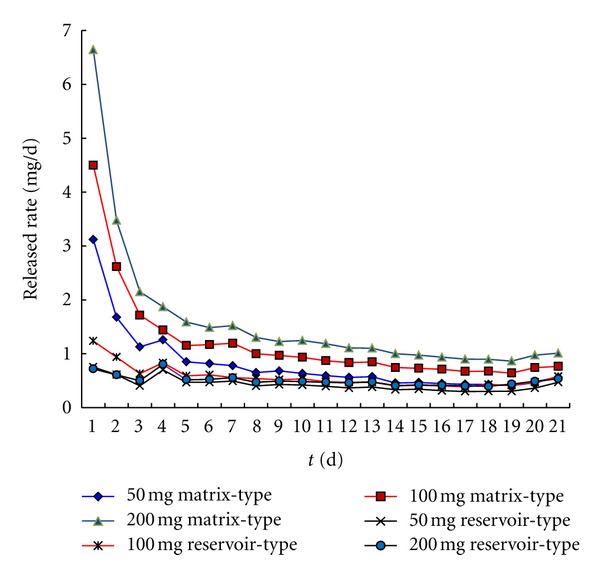
The influence of matrix-type and reservoir-type IVR on the release profile of drospirenone.

The mean daily release profiles of drospirenone from matrix IVR and reservoir-type devices, loaded with 50 mg, 100 mg, and 200 mg, are shown in Figure 7. As can be seen for the matrix-type IVR, a relatively large amount of the drug permeates from the device during the initial release period (a “burst effect”) followed by a decreased steady-state level thereafter. The initial burst continues for the first 2 days of release, although the day 1 burst is substantially larger than for day 2. The magnitude of the day 1 bursts and the subsequent steady-state fluxes (mean daily release, mg/day) were increasing with drospirenone content increasing from 50 mg to 200 mg. However, concerning the release profiles of reservoir-type systems, no significant “burst effect” has been observed and the cumulative drospirenone release obtained was linear with time verifying a zero-order release rate (Figure 10(b), Table 4) with corresponding decreasing daily release rate for all dosage level compared with matrix type. The result also confirmed that it is important to note that the coating and layering prevented the initial burst effect and provided a fairly stable and efficacious release of drug from the ring [10].

**Figure 8:**
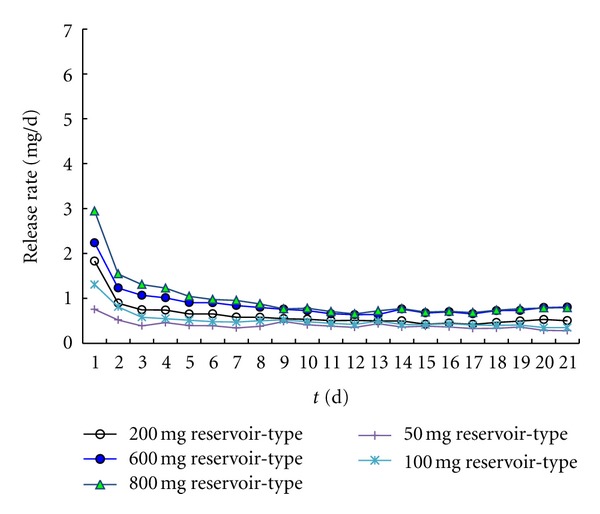
The influence of the content of drug on the release profile of drospirenone reservoir-type IVR.

Due to the characterization of decreasing the “burst effect” and a zero-order release rate for the reservoir-type, the drug content added from 50 mg to 800 mg has been investigated in this kind of type VCR. From the perspective of process, 800 mg loaded was observed us maximum loading in the silicon elastomer. For a given core/sheath ratio, the only influence of increasing drug loading should be to prolong the duration of constant daily release. As can be seen from Figure 8, the release rate was no more increasing with 800 mg compared with 600 mg.

From release profile of the matrix type and reservoir type, release flux after 3-day could keep constant rate. A rationale for zero-order release can be the low partition coefficient of drospirenone between the polymer and the sink conditions utilized in the release studies. According to the modeling performed by Chien et al. [12], it is possible to obtain a zero-order release from this system in cases where the thickness of the drug depletion zone is small owing to a small partition coefficient of the drug between the polymer and the sink.

This reservoir type offers advantage over the matrix type by the absence of burst release, but needs higher drug loading requirements. Therefore, increasing the release rate of drug with lower drug loading was to be investigated as follows.

**Figure 9:**
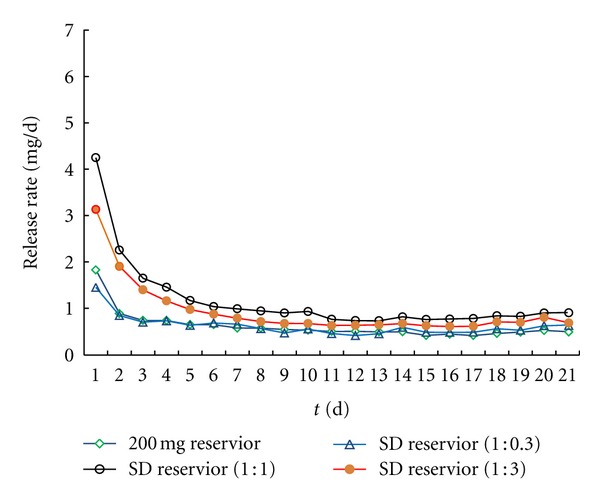
The influence of the PVP solid dispersion (SD) in reservoir-type IVR on the release of drospirenone.

As paper [13] reported, the additives putting into polymer formulation could increase daily rate. The hydrophilic excipient selected was PVP_k30_ in the present study. The incorporation of PVP into the IVRs had no effect of release. However, drospirenone converted into solid dispersion form with PVP_k30_ carrier, and the result had an effect of increasing the release rate while slightly increasing the magnitude burst (Figure 9 and Table 4). The release rates were not noted to increase linearly with increasing PVP_K30_ loading (Figure 9), while the proportion of PVP and drug (1 : 1) had better result.

The incorporation of PVP_k30_ into the IVRs had no effect of release, which is therefore considered as that the incorporation of PVP into the cross linked elastomer network did not provide a more “open” network structure leading to enhanced release rate [13]. The hydrophobic properties might not be modified through the addition of hydrophilic substances PVP into the elastomer mix prior to cross linking/molding as the paper reported. Silicone elastomers are hydrophobic materials with excellent water-repellent characteristics. It is also postulated that solubility of drospirenone has an increase in the PVP-SD, while solubility decreases in the silicone elastomer network. However, this method was particularly useful in enhancing the release of hydrophobic drugs in the silicon elastomer [14].

**Figure 10:**
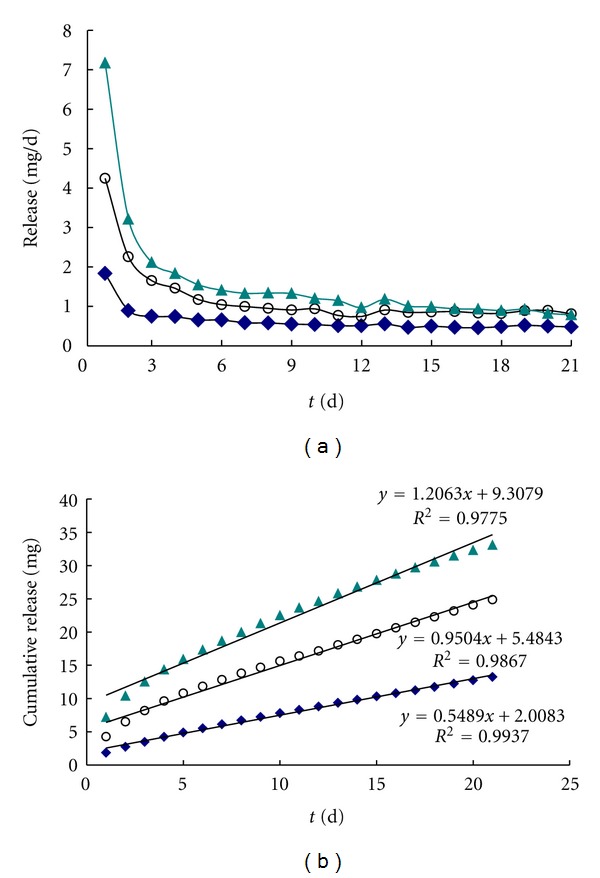
Mean *in vitro* daily (a) and cumulative (b) release of profile of drospirenone from different type silicone vaginal rings (▲ matrix type; ○ PVP-SD (1 : 1) reservoir type; ◆ reservoir type) loading the same weight 200 mg drug in 21 d.

In order to compare the different types of VCR studied in this paper, the cumulative release of different type of drospirenone IVR was fitted to zero release equation, and the cumulative release equations based on day 1 to day 21 data, along with respective *r* coefficient are presented in Table 4. Compared with release profile of matrix-type, reservoir-type and PVP-SD reservoir loading 200 mg drospirenone, (Figure 10), the daily release of reservoir system of drospirenone IVR was about 0.5 mg, which sustained 21 days at this release rate and significantly decreased burst effect compared with matrix system. When drospirenone is modified with PVP_k30_ in the reservoir system formulation, the daily release quantity increased and sustained 21 days at the release rate of about 1.0 mg/d, which had a decreasing burst effect and constant rate compared with matrix (Table 4: burst = 5.04843, 9.3079; *r* = 0.9932, 0.9896).

Extrapolating the cumulative flux to the matrix, reservoir-type ring, and PVP-SD reservoir-type ring loaded 200 mg shown in Figure 10, we achieved roughly 0.5, 1.0 mg drospirenone daily release during 21 days with low burst effect. It is uncertain what quantity of drospirenone should be released from the IVR *in vitro* since a disparity often exists between *in vitro* release and *in vivo* vaginal release and subsequent biodistribution. For these reasons, controlled release is critical to the formulation of drospirenone IVR. Given that drospirenone shows initial loading-dependent release in our formulation *in vitro*, the loading can likely be adjusted to release an amount of drug that is safe and deemed adequate to prevent pregnancy in humans. Furthermore, our IVR design allows for adjustment of the PVP content.

This system offers advantage over the reservoir system by having lower drug loading requirements and the absence of burst release. The release rate of drug was observed to be proportional to the drug loading in a certain range.

Although the release rate can be modified by changing the initial loading, this might also change the duration of desired release rate for the system. However, we have demonstrated that sustained zero-order release of drug can be obtained successfully from rings ranging from 0.3 to 1.0 mg/day, depending on drug loading.

### 3.6. Compatibility Study of API and Excipients

The compatibility of drospirenone with PVP_k30_ and/or silicone elastomer was tested respectively under the condition of 60°C, relative humidity of 92.5%, and illuminated at 4500 Lx, respectively, for 5 d and 10 d, at which time drug extraction and analysis was performed. Changes of impurities (single impurities and total impurities) from final treated silicon elastomer ring were extracted by solvent, detected by HPLC, and calculated as area percent (%) of total peak area (Tables 5, 6, and 7).

We studied drug degradation using high temperature and humidity conditions since this would be indicative of long-term drug degradation under ambient conditions and may suggest possible degradation mechanisms. The results indicate no significant degradation of drospirenone in polymer at 5 and 10 days when compared to day 0. In addition, there were no new peaks in the sample chromatograms that would indicate possible degradation. Altogether, these results indicate that drospirenone is stable in their respective PVP or silicon elastomer. The results of this study demonstrate that drospirenone might be stably formulated into silicon elastomer intravaginal ring for 21-day sustained release.

### 3.7. Tolerability of Drospirenone IVR in Tissue Level

**Figure 11:**
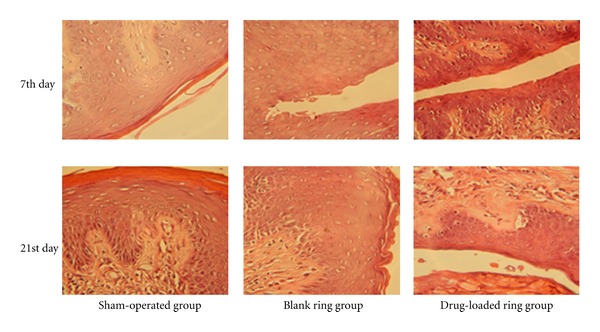
Morphology of vaginal tissues after application of VCR containing DRSP. Drospirenone IVR cut into rod about 1 cm length was administered into the vagina of the rats. The vaginal tissues of three different experimental groups (*n* = 10 each) were used after 7th day and 21st day: sham-operated animals with normal diet (control group); blank reservoir ring administrated group (blank group); drug-loaded reservoir-type ring administrated group (experimental group) was isolated, fixed in 10% neutral carbonated-buffered formaldehyde, embedded in paraffin, and cut into slices. After hematoxylin-eosin staining, the slices were observed under a light microscope (×400).

IVR did not alter the morphology of vaginal tissues. Figure 11 showed the histopathology of the vaginal mucosa after intravaginal application of IVR. As compared to the control with no treatment, the IVR-treated group showed no visible sign of inflammation or necrosis. IVR did not affect the morphology of vaginal tissues, which indicated that IVR device was being with good biocompatibility and such drug delivery systems might be safe for vaginal delivery.

## 4. Conclusions

Intravaginal ring (IVR) containing drospirenone is one kind of vaginal ring devices, and the drug-loaded core was obtained by using hot vulcanization method with silicone elastomer as the matrix polymer, which was encapsulated by the same kind of polymer membrane. A method for this preparation of drospirenone reservoir system was obtained by adding PVP_k30_ additives to increase drospirenone solubility. Drospirenone was used as the active pharmaceutical ingredient and silicone elastomer as matrix and controlled release membrane agent. PVP_k30_ was used as increasing drospirenone solubility and without resulting in the drospirenone degradation detected by HPLC gradient method. Under strong light, high temperature, and high humidity conditions, drospirenone raw materials and formulation excipients PVP_k30_ and silicon rubber had no significant interactions and indicate good compatibility. It is found that the drospirenone release rate was increasing with the PVP_k30_ amount at a constant range. Silicone elastomer was also employed as membrane for controlling membrane permeability. *In vitro* drug release testing was conducted in a dissolution apparatus, the release medium was 200 mL 0.3% SDS meeting with sink conditions, and release samples were determined by the UV-Vis spectrophotometry. The daily release of reservoir system of drospirenone IVR was about 0.5 mg, which sustained 21 days at this release rate and significantly decreased burst effect compared with matrix system. When the drug is modified by PVP_k30_ in the reservoir system formulation, the daily release quantity increased and sustained 21-days at the release rate of 1.0 mg/d. The cumulative release of reservoir of drospirenone IVR was fitted to zero release equation. In addition, drospirenone IVR system was evaluated by tolerability on tissue level in rat. Drospirenone IVR did not affect the morphology of vaginal tissues.

Therefore, the dosage form might be further developed for safe, convenient, and effective contraceptive drug delivery with reduced dosing interval. The results of this study demonstrate that drospirenone may be stably formulated into silicon elastomer intravaginal ring for 21-day sustained release.

## Figures and Tables

**Table 1:** HPLC conditions.

Column	Diamonsil C_18_ (250 mm × 4.6 mm, 5 *μ*m)
Column temperature	Ambient temperature
Mobile phase	Water: acetonitrile (gradient elution process, see Table 2)
Flow rate	1.5 mL/min
Injection volume	10 *μ*L
Detection	245 nm
Apparatus	Waters 2695
Runtime	80 min
Retention time	About 32 min

**Table 2:** The gradient elution process for HPLC of drospirenone.

*T* (min)	Water (%)	Acetonitrile (%)	Elution
0–28.5	64	36	isocratic
28.5–45	64–100	36–90	Linear gradient
45–45.5	10–0	90–100	Linear gradient
45.5–52	0	100	isocratic
52-53	0–64	100–36	Linear gradient
53–80	64	36	Reequilibration

**Table 3:** Thermal parameters obtained by differential thermal scanning calorimetry of drospirenone in silicone elastomer (*n* = 3, mean ± SD).

Drospirenone concentration (%)	Enthalpy of melting Δ*H* (J/g)	Onset temperature (°C)	Peak temperature (°C)
1.0	0.34 ± 0.04	191.50 ± 0.26	193.70 ± 0.20
2.0	0.94 ± 0.07	191.82 ± 0.25	193.80 ± 0.02
5.0	2.52 ± 0.07	192.39 ± 0.43	194.61 ± 0.37
10.0	4.81 ± 0.09	192.35 ± 0.39	194.52 ± 0.59
15.0	7.53 ± 0.20	192.15 ± 0.19	194.47 ± 0.25
20.0	8.83 ± 0.15	192.46 ± 0.23	195.07 ± 0.45

**Table 4:** Summary of the release data for the matrix-type, reservoir-type, and PVP-SD-treated reservoir IVR containing drospirenone.

Type	Equation of line of best fit	(*r*) coefficient	Release rate (mg/day)	Burst (mg)
Matrix type	*y* = 1.2063*x* + 9.3079	*r* = 0.9896	1.2063	9.3079
Reservoir type	*y* = 0.5489*x* + 2.0083	*r* = 0.9968	0.5489	2.0083
PVP-SD reservoir IVR	*y* = 0.9504*x* + 5.04843	*r* = 0.9932	0.9504	5.04843

PVP-SD: drospirenone solid dispersion with PVP_k30_ as carrier.

**Table 5:** The results of API excipients compatibility testing under strong light exposure (4500 LX).

Light exposure	API and excipients		
	Drospirenone	Drospirenone + PVP_k30_	Drospirenone + silicone elastomer
0 d			
S (%)	0.03	0.08	0.03
T (%)	0.09	0.16	0.14
5 d			
S (%)	0.03	0.14	0.03
T (%)	0.11	0.22	0.17
10 d			
S (%)	0.08	0.15	0.03
T (%)	0.16	0.22	0.15

S: single impurities; T: total impurities; API: drospirenone.

**Table 6:** The results of API excipients compatibility testing under high temperature.

High temperature (60°C)	API and excipients		
	Drospirenone	Drospirenone + PVP_k30_	Drospirenone + silicone elastomer
0 d			
S (%)	0.03	0.08	0.03
T (%)	0.09	0.16	0.14
5 d			
S (%)	0.02	0.03	0.03
T (%)	0.08	0.08	0.15
10 d			
S (%)	0.08	0.08	0.03
T (%)	0.28	0.29	0.13

S: single impurities; T: total impurities; API: drospirenone.

**Table 7:** The results of API excipients compatibility testing under high humidity.

RH 92.5%	API and excipients		
	Drospirenone	Drospirenone + PVP_K30_	Drospirenone + silicone elastomer
0 d			
S (%)	0.03	0.08	0.03
T (%)	0.09	0.16	0.14
5 d			
S (%)	0.02	0.03	0.03
T (%)	0.09	0.06	0.13
10 d			
S (%)	0.07	0.08	0.03
T (%)	0.14	0.30	0.15

S: single impurities; T: total impurities; API: drospirenone.
